# Undergraduate complete denture education in transition: balancing conventional and digital workflows

**DOI:** 10.3389/froh.2026.1874471

**Published:** 2026-07-06

**Authors:** Mohammad Majduddin Sulaiman, Nur Haneena Abdul Halim

**Affiliations:** 1 School of Dental Sciences, Universiti Sains Malaysia, Kota Bharu, Malaysia; 2 Klinik Rawatan Keluarga Pergigian, Hospital Pakar Universiti Sains Malaysia, Kota Bharu, Malaysia

**Keywords:** complete denture, digital denture, digital workflow, undergraduate dental curriculum, undergraduate dental education

## Introduction

Complete denture prosthodontics is still a part of the undergraduate dental curriculum. The teaching has been based on conventional methods such as impression procedures, jaw relation records and tooth arrangement. But the rapid rise of digital denture workflows using computer-aided design and manufacturing (CAD-CAM) is changing clinical practice. The challenge for dental educators now is to fit these innovations into an already limited curricular time. The question is no longer whether complete denture digital workflows should be taught but how we can integrate them without sacrificing needed clinical competencies.

## Discussion

Conventional complete denture procedures remain foundational because they foster an understanding of the biological and functional principles that govern successful prosthodontic treatment. Border moulding, definitive impressions and recording maxillomandibular relationships are important procedures for retention, stability and support of complete dentures. More importantly, these procedures also teach students about the behaviour of oral mucosa under functional loading, how neuromuscular control influences denture stability, and how errors in clinical records can be propagated through treatment.

While digital workflows can facilitate some aspects of denture fabrication, they do not replace the biological principles that determine treatment success. Students lacking a deep understanding of conventional techniques may perceive complete denture fabrication as merely a technical process rather than a biologically driven rehabilitative therapy.

Advantages of digital denture workflows are reduced clinical visits, standardised fabrication and improved reproducibility (1). The retrospective study by Casucci et al. (1) showed improvements in workflows efficiency and cost effectiveness associated with digital complete denture fabrication. While these outcomes may improve clinical productivity, they do not necessarily reduce the educational need for conventional denture fabrication. Intraoral scanning, virtual articulation and CAD-CAM manufacturing are increasingly integrated into current prosthodontic practice. Consequently, familiarity with such technologies is becoming increasingly important for new dental graduates entering clinical practice.

Bringing in digital workflows at undergraduate level can help to raise awareness of contemporary practice and adaptability to emerging technologies. Importantly, exposure to digital workflows also offers opportunities to critically discuss the limitations of digital data acquisition. The scoping review by Achmadi et al. (2) highlighted ongoing challenges in accurately recording the edentulous ridge using intraoral scanners, particularly in recording mobile soft tissues, peripheral extensions, and functional sulcus depth. These limitations are of educational significance because they demonstrate that digital impressions are still dependent on biological interpretation and clinical judgment rather than only technical image acquisition. Digital workflows should be taught with a strong prosthodontic foundation, including indications, limitations and decision making, and not just the technical execution.

Undergraduate dental curricula are constrained by time and resources and the necessity to ensure competence across disciplines (3). Hence, equal depth of training in conventional and digital denture fabrication may not be realistic within current educational structures. Also, the availability of digital infrastructure and trained faculty vary greatly between dental institutions (4). These constraints necessitate a pragmatic and prioritised curricular approach to complete denture education.

A pragmatic educational approach is therefore to prioritise competence in conventional complete denture procedures while incorporating structured exposure to digital workflows. A staged hybrid curriculum model may therefore represent the most appropriate educational approach for undergraduate denture education. Early preclinical teaching should continue to focus on conventional complete denture principles. Structured digital workflows exposure can then be introduced gradually in the later preclinical and clinical years through lectures, simulation-based demonstrations, digital laboratory observations and limited supervised hands-on experiences (5). The goal is not to produce experts in digital workflows but to equip students with the ability to critically appraise digital technologies, identify appropriate clinical indications and participate in supervised denture digital workflows within clinical practice (4). Graduates should be able to evaluate different treatment pathways and select appropriate workflows considering patient-specific factors.

## Conclusion

Digital workflows in complete denture are likely to become increasingly common, but their incorporation should complement rather than replace conventional undergraduate teaching. A hybrid educational model is therefore required to preserve core prosthodontic competence while preparing graduates for evolving digital practice. By building a robust foundation of conventional principles and incorporating digital concepts, dental schools can produce graduates who are clinically competent and capable of adapting to technological change. The goal of undergraduate education is not mastery of a specific workflow but the development of good clinical judgment. In this regard, the balance of conventional and digital denture teaching is a necessary future-oriented educational strategy.
